# Amino Acid Sensing *via* General Control Nonderepressible-2 Kinase and Immunological Programming

**DOI:** 10.3389/fimmu.2017.01719

**Published:** 2017-12-11

**Authors:** Srikanth Battu, Gillipsie Minhas, Aman Mishra, Nooruddin Khan

**Affiliations:** ^1^Department of Biotechnology and Bioinformatics, School of Life Sciences, University of Hyderabad, Hyderabad, India; ^2^Department of Biomedical Engineering, IIT Hyderabad, Hyderabad, India

**Keywords:** amino acid sensing, general control nonderepressible-2 kinase, mammalian target of rapamycin, inflammation, immune response, metabolic diseases

## Abstract

Metabolic adaptation to the changing nutrient levels in the cellular microenvironment plays a decisive role in the maintenance of homeostasis. Eukaryotic cells are equipped with nutrient sensors, which sense the fluctuating nutrients levels and accordingly program the cellular machinery to mount an appropriate response. Nutrients including amino acids play a vital role in maintaining cellular homeostasis. Therefore, over the evolution, different species have developed diverse mechanisms to detect amino acids abundance or scarcity. Immune responses have been known to be closely associated with the cellular metabolism especially amino acid sensing pathway, which influences innate as well as adaptive immune-effector functions. Thus, exploring the cross-talk between amino acid sensing mechanisms and immune responses in disease as well as in normal physiological conditions might open up avenues to explore how this association can be exploited to tailor immunological functions toward the design of better therapeutics for controlling metabolic diseases. In this review, we discuss the advances in the knowledge of various amino acid sensing pathways including general control nonderepressible-2 kinase in the control of inflammation and metabolic diseases.

## Introduction

The association between inflammation and metabolism has been known since 1930s when Kempner and Peschel published their work on the subject and used the term “immunometabolism” for the first time (1, 2). However, growing evidence suggests that the nutrient availability and cellular metabolism plays a central role in physiological processes including cell proliferation, differentiation, and cell death (3). Accordingly, all organisms have developed diverse sensing mechanisms to detect and respond to scarcity and abundance of different nutrients. Unlike unicellular organisms (which sense the availability of nutrients in the environment directly), multicellular organisms consist of various nutrient sensing mechanisms to sense and respond to both extracellular and intracellular nutrient fluctuations (4, 5). Eukaryotic cells detect changes in nutrients levels through sensors that could be a transporter, receptor, signaling proteins or an enzyme. This is also pertinent to the immune cells, as they highly rely on energy supplies for expansion, differentiation, and the synthesis of immune effector molecules required for clearance of the infected or altered cells (3). The close association of immune system with the metabolic pathways involved in the sensing, management of the metabolites plays a crucial role in the maintenance of immune homeostasis, and therefore, dysregulation in the functioning of these pathways results in chronic inflammatory conditions (3). Accumulating evidence suggests that caloric restriction (CR) without malnutrition increases lifespan and protects from age-associated inflammatory diseases, such as diabetes, cardiovascular diseases, cancer, and brain atrophy (6, 7). It has been postulated that regulatory genes, which are fundamental to energy metabolism play a vital role in CR-induced physiological benefits (8). Although CR-associated benefits have often been related to reduced energy intake, growing shreds of evidence implicate dietary amino acid limitations to CR benefits (9). Amino acids are the building blocks of proteins and the predominant macromolecules in the cell. Also, amino acids are vital nutrients for cellular homeostasis, not only as the energy source or constituents of proteins but also as the signaling modules, which is evident from the evolutionarily conserved pathways that play a fundamental role in amino acid sensing (10). Recent studies have unveiled that amino acid restriction alone enhances insulin sensitivity in mice (11). Also, it has been shown that administration of reduced amino acid diet protects mice from DSS induced intestinal inflammation by activating the cellular homeostatic process, such as autophagy (12). Therefore, understanding the mechanisms through which cells sense and mount an appropriate response to the bioavailability of amino acids has been an area of active research. It has been well established that amino acids presence or absence in mammals are sensed by predestined distinct signaling pathway involving mammalian target of rapamycin (mTOR) or general control nonderepressible-2 kinase (GCN2), respectively (13). mTOR acts as one of the primary metabolic switches, which integrate amino acid, growth factor, and energy availability to promote anabolic processes such as protein synthesis, while at the same time it inhibits catabolic functions such as autophagy (14). In contrary, depletion of even single amino is directly sensed by GCN2, which in turn program cellular machinery to promote catabolic processes such as autophagy. Recent studies implicate the centrality of GCN2 in the maintenance of immune homeostasis (15), and the dysfunction underlies several chronic metabolic diseases (12, 16). Here, we present a survey of the current research advances made toward understanding the link between GCN2-mediated amino acid sensing mechanisms and immunological regulation and further discuss the recent findings of their implications in the pathogenesis of acute or chronic inflammatory and metabolic diseases.

## Amino Acid Sensing Mechanisms

**Figure 1:**
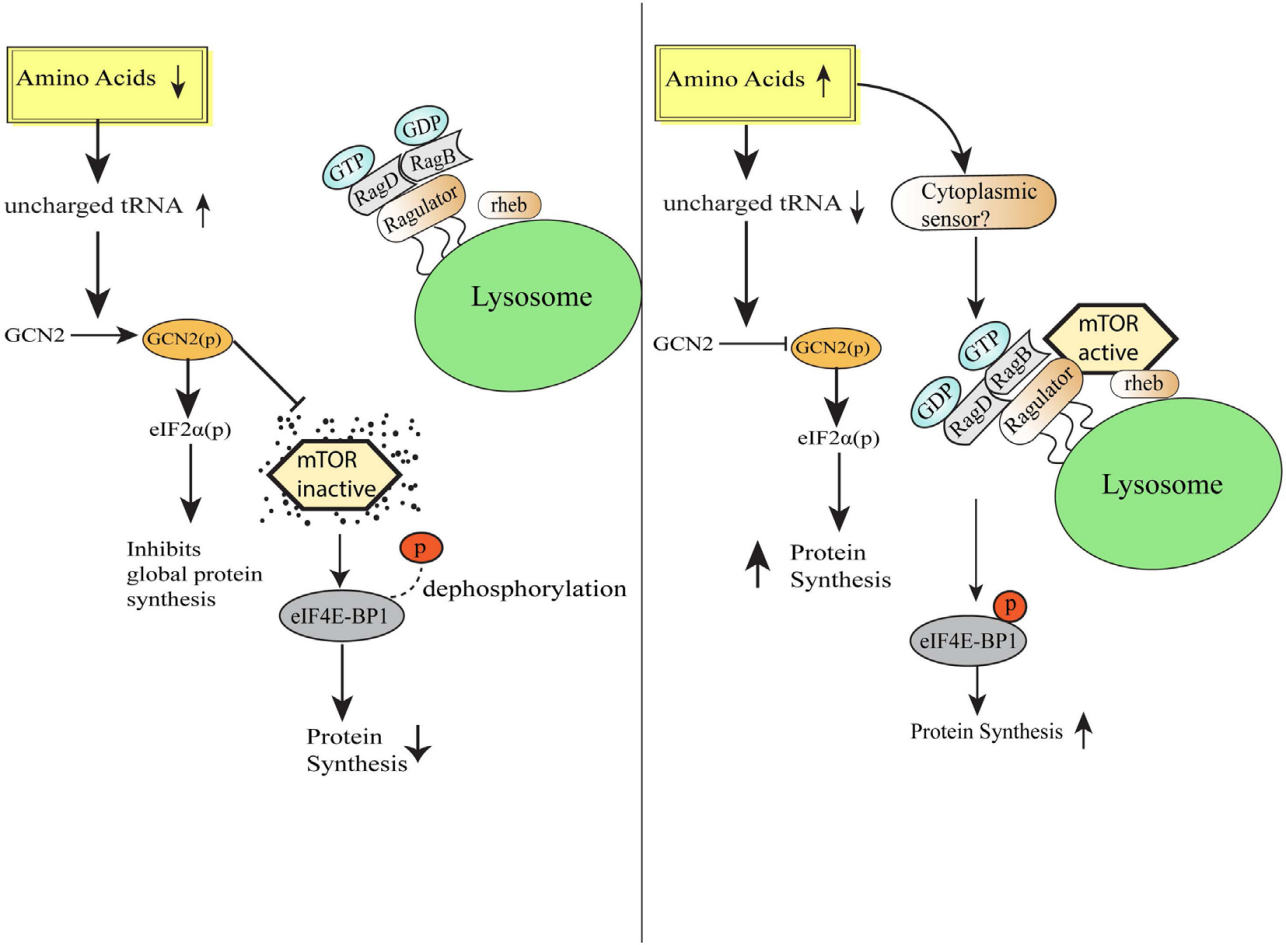
Amino acid sensing and integration of downstream pathways. Schematic representation of the cellular events during amino acid-deficient and amino acid sufficient conditions. General control nonderepressible-2 kinase senses amino acid insufficiency and orchestrate various homeostatic processes *via* eIF2 phosphorylation followed by downregulation of global protein synthesis and simultaneously also inhibits mammalian target of rapamycin (mTOR) activation. On the other hand, under the condition of amino acid sufficiency, mTORC1 complex translocates to lysosomal surfaces by virtue of Rag GTPase activation and further initiates protein translation by the release of translation initiation factor eIF4E.

Amino acids are the vital macronutrients that not only serve as the primary building blocks of proteins but also serve as an alternate energy source (17). The process of protein synthesis is one of the most energy-requiring processes in the cell, and therefore, mechanisms to efficiently sense the availability of amino acids and trigger appropriate responses become pertinent for the maintenance of cellular homeostasis (18). Across the species, various mechanisms have been evolved to detect the scarcity or abundance of different extracellular and intracellular amino acids in the microenvironment. Bacterial cells sense amino acid bioavailability through programming its transcriptional control, while eukaryotic cells sense footprints of amino acids scarcity by diverse mechanisms, including accumulated uncharged cognate tRNAs sensing (9). Eukaryotic cells are well equipped with sensors such as mTOR, which gets activated during the amino acid sufficiency and programs various anabolic processes required for the growth (19, 20). In contrast, intracellular depletion of even single essential amino acid (EAA) or non-essential amino acid is directly sensed by the GCN2 *via* binding of uncharged cognate tRNAs (21). GCN2 and mTOR pathways have evolved together in eukaryotes to serve as a major regulatory switch dictating protein synthesis in response to the fluctuating levels of amino acids (22). mTOR, an evolutionarily conserved serine/threonine kinase initially identified in yeast as TOR (23), is activated in the presence of specific amino acids especially leucine, arginine, and methionine (19, 20) and links amino acids availability with the cell growth, proliferation, and differentiation (24–26). Accumulating evidence suggests that mTOR localizes to lysosomes as a function of amino acids (27). During amino acid sufficiency, vacular H^+^ATPase (v-ATPase, the first downstream target known so far) triggers the guanine exchange factor activity of Ragulator complex, which results in the nucleotide exchange and activation of RAG GTPase (28). Further, activated RAG GTPase binds and recruits mTORC1 to the lysosomal membrane in close proximity to mTORC1 activator RHEB (27, 29). Together, these stimuli lead to the mTORC1 activation. Activated mTORC1 translocates to the lysosome and phosphorylates 4EBP1, to release the translation initiation factor, eIF4E, which recruits mRNA to the ribosomes to initiate protein synthesis (24, 30) (Figure 1). Also, mTOR has central control over various transcription factors, like NF-κB, STAT3, and HIF1α (31). Conversely, in the absence of amino acids, mTOR is inactivated and diffused in the cytosol (32), which increases the 4EBP1 de-phosphorylation and halts protein translation (20). Albeit the precise amino acid sensor in the cytosol or at the lysosome is unknown, recent cell-based biochemical studies have shown the proteins responsible for Rag GTPases tethering to the lysosomal surfaces (27), and other regulatory proteins functioning upstream of Rag GTPases (28, 33, 34).

Mammalian target of rapamycin integrates various cellular functions including protein synthesis, cell proliferation, autophagy, and metabolism. It gets activated by virtue of signaling events initiated by receptors for particular antigens, cytokines, and growth factors (35). Several studies report that antigen engagement of T-cell receptor (TCR) and CD28 (costimulatory receptor) leads to the activation of phosphatidyl inositol 3 kinase (PI3K) and Akt, which eventually leads to mTOR activation (36, 37). Albeit Akt plays a central regulatory role in signaling pathways implicated in T cell proliferation, metabolism, migration, and activation (38, 39), a study has demonstrated that phosphorylation of S6 downstream of the TCR and CD28 stimulation is not majorly dependent on Akt (40). Furthermore, a recent study by Hamilton et al., established that adaptor protein Carma1 and one of its associated proteins, MALT1 are essential for optimal activation of mTOR in T cells (41). Also, Akt and mTOR pathways play a key role B cell proliferation and differentiation (42).

Recent studies suggest that mTOR gets activated in immune cells through numerous factors including growth factors, cytokines, and TLR ligands association with its cognate receptor. Activation of the receptor leads to the recruitment of PI3k to the receptor complex *via* various adaptor molecules including the GTPase RAB8A. Further PI3K induced secondary messenger phosphatidylinositol-3,4,5-trisphosphate recruits and activates Akt, which consists of two key effectors such as FOXO1 and TSC2. In unstimulated cells, TSC2 heterodimerizes with TS1 and causes mTOR inactivation. Conversely, stimulation of cells results in TSC2 phosphorylation at threonine 1462 by Akt, which further leads to mTOR activation eventually (43).

On the other hand, general control nonderepressible 2 kinase (GCN2), a serine/threonine kinase, which detects the scarcity of any amino acids and constitutes the evolutionarily conserved amino acid starvation response (AAR) pathway. Under normal physiology, during protein translation, amino acid-loaded tRNAs assemble at ribosome and provide amino acids for the elongation of nascent peptide. During amino acid deficiency, uncharged or unloaded tRNAs accumulate in the cell and initiate signaling pathways to reserve cellular energy and resources by repressing global protein translation, at the same time derepressing the translation of particular mRNAs required for the restoration of cell homeostasis (44). During amino acid deficiency, accumulated tRNAs bind to the GCN2 kinase, which undergoes a conformational change to initiate a downstream pathway. Basically, binding of GCN2 kinase with uncharged tRNAs initiates autophosphorylation (due to the conformational change) and simultaneous phosphorylation of translation initiation factor, eIF2α at serine 51, which results in the attenuation of general protein translation initiation due to decrease in eIF2/tRNAiMet/GTP ternary complex, required for polysome formation and translation (45) (Figure 1). Thus, translationally stalled mRNAs along with initiation factors assemble into stress granules (SGs) through the recruitment of RNA-binding proteins (RBPs), such as TIA-1/TIAR, which in turn determines the fate of mRNAs translatability or decay (46). Simultaneously, translation of specific stress-responsive mRNAs, such as the transcription factors ATF4 and CHOP that influence the genes involved in the amino acid synthesis, homeostasis, and cell survival are upregulated (47).

General control nonderepressible-2 kinase is among the four vital kinases along with PKR, PERK, HRI which form the core of integrated stress response (ISR) pathway (46). While GCN2 is a metabolic sensor, HRI senses heme deprivation within the cell, PERK senses ER stress, and PKR gets activated as a part of the antiviral response of mammalian cell by detecting viral dsRNA, interferons (IFNs), and growth factors (26). All these four kinases upon activation phosphorylate eIF2α, which in turn decreases the global proteome of the cell as an artistry to economize the energy of the cell for the maintenance of cellular homeostasis (48, 49). Earlier studies report a cross-talk between the GCN2 and mTOR signaling pathways in both yeast and mammals (50, 51). Further, through mixed lymphocyte reaction in human CD4^+^ T cells, Eleftheriadis et al., demonstrated that both these pathways elicit immunosuppression, influence cell proliferation, and differentiation, but through different mechanisms (52). Moreover, in a study by Xiao et al., the authors reported that dietary leucine deprivation enhances the insulin sensitivity in mice (11). Similarly, pharmacological or genetic reduction in mTOR signaling has been shown to increase the lifespan in model mammalian organisms. Consequently, it becomes inevitable to gain the better understanding of the role of amino acid sensing pathways in immunomodulation and how it can be used to design potential therapeutic targets for metabolic diseases.

## Amino Acid Sensing by GCN2 and Inflammation

The initiation of inflammation during any infection/injury is characterized by the recruitment of neutrophils and other immune cells at the site of pathogen invasion (53) followed by the release of a storm of cytokines, chemokines, and other immune effector molecules. As the pathogen is cleared, resolution phase of inflammation sets in, that is marked by tissue homeostasis and regeneration (54). All these processes require enormous energy and nutrients especially amino acids.

**Figure 2:**
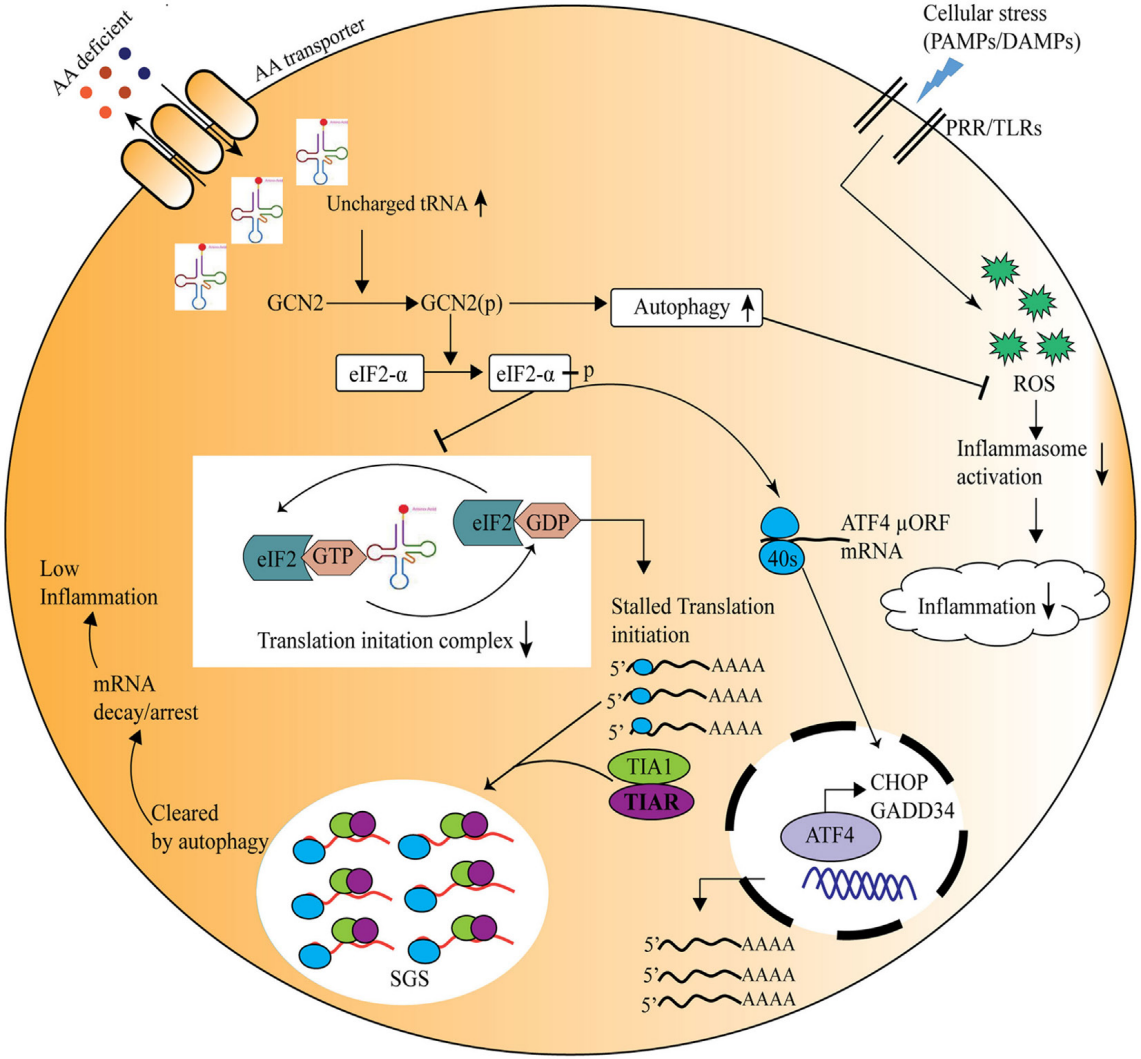
General control nonderepressible-2 kinase (GCN2)-mediated amino acid sensing in the control of inflammation during cellular stress. Amino acid deficiency leads to accumulation of uncharged tRNAs, which are recognized by the nutrient sensor GCN2. Upon its activation, the phospho-GCN2 inhibits the translation initiation ternary complex through the phosphorylation of eIF2α, thus stalls the protein translation. As a result, the RNA-binding proteins are recruited to the translationally stalled mRNAs, forming stress granules that either arrest or decay the stalled mRNAs, and ultimately decreasing the inflammation. At the same time eIF2 phosphorylation leads to translation of specific mRNA, such as ATF4, which translates stress-response genes, GADD34, CHOP, etc. On the other hand, GCN2 pathway induces autophagy which reduces inflammation through inhibition of the reactive oxygen species and inflammasome activation.

In recent times, burgeoning information about the amino acid sensing pathways and their link to innate as well as adaptive immunity has emerged (55). Immune cells are known to be auxotrophs for amino acids, and the condition of inflammation or infection influences cellular amino acid requirement for protein synthesis (56). During an immune response, a redistribution of amino acids is observed, with a shift from cellular growth to the synthesis of immunological proteins (57). Lately, it has also become evident that local deprivation of amino acids could modulate immune responses (58). For instance, during pathogen invasion, such as *Shigella* infection, the bacteria damage the host cell membrane and create an environment of amino acid scarcity, which induces the GCN2 kinase-mediated activation of ISR pathway, and simultaneously reduction in mTOR activity (59). Thus, host senses the amino acid starvation induced by pathogens and triggers protective immune responses. It has also been shown that immune response activated under the conditions of starvation severely affects the survival of insects (60). Further insights into the mechanisms implicated in amino acid sensing driven protective responses identified a crucial role for the amino acid sensor, GCN2 in the metabolic reprogramming in response to cellular stress (61). In *Drosophila*, ingestion of *Pseudomonas entomophila* damages the gut and inhibits translation *via* GCN2 kinase activation and TOR inhibition. The inhibition of protein translation not only leads to the shaping of immune response but also in pathogenesis, by inhibiting repair processes (62). Concomitantly, mice deficient in GCN2 are highly prone to intestinal inflammation, while activation of the GCN2 pathway by reduced amino acid diet dramatically reduced inflammation-associated intestinal pathologies (12). During amino acid insufficiency, uncharged tRNAs accumulate and bind to GCN2 which results in a conformational change in GCN2 and its kinase activation (4). Activated GCN2 phosphorylates translation initiation factor eIF2α which further leads to decrease in ternary complex, such as eIF2/tRNAiMet/GTP, essential for translational initiation (45). Thus, 48S preinitiation complex assembly at 5′ region of capped mRNA is interrupted, which leads to polysome disassembly and SG formation through recruitment of RBPs, such as TIA-1/TIAR (45) (Figure 2). These RBPs specifically bind to adenine- and uridine-rich elements present at 3′ untranslated regions of mRNAs through RNA recognition motif and dictate their stability/degradation (63). In immune cells, RBPs play a central role in the posttranscriptional regulation of immune effector molecules. It has been shown that RBPs, HUR, and TIA-1 act together and inhibit the translation of proinflammatory cytokines such as TNF and IL-1β thereby offer anti-inflammatory effect (63). In addition to SGs formation, amino acid deprivation activates autophagy (a cellular stress response that targets long-lived proteins, damaged cell organelles to lysosomes for degradation and is known to affect inflammation negatively) (64, 65) to maintain cellular homeostasis. In a recent study Ravindran et al. show that mice deficient of GCN2 produced substantially high level of reactive oxygen species (ROS) and subsequently proinflammatory mediator, IL-1β, in response to cellular stress, where they suggest that this effect is due to lack of autophagy in GCN2-deficient mice, whereas reduced amino acid diet fed mice show significantly low level of oxidative stress and inflammatory responses to cellular stress (12). Further mechanistic insights depict that GCN2 induced autophagy interferes with oxidative stress and inflammasome activation thereby controls inflammation (12) (Figure 2). Furthermore, Liu et al., demonstrate that GCN2 kinase signaling significantly affect macrophage cytokine production in response to LPS stimulation of macrophages expressing indolamine 2,3-dioxygenase (IDO), and further suggest that although GCN2 mediates the enhanced IL-6 mRNA expression, its translation is blocked by virtue of eIF2 phosphorylation (61, 66).

## GCN2-Mediated Amino Acid Sensing and T-Cell Immune Response

T cells constitute the major component of adaptive immunity and are prevalent during inflammation, infection, and autoimmune diseases. αβT cells are classified majorly based on the coreceptor as CD8^+^ and CD4^+^ (67). Upon activation of naive or resting T cell by cognate antigen-MHC signals, they undergo rapid expansion, differentiation followed by contraction (68). CD8^+^ T cells expand and differentiate into effector cytotoxic cells, which help in clearing tumor, virally infected cells (69), while CD4^+^ cells undergo proliferation and functional specialization, depending upon the cytokine milieu, as Th1, Th2, Th17, or regulatory T cell (Treg) response, each with its effector function (70). T cell population undergoes different stages of development in the thymus to activation, expansion, differentiation, and migration at the site of requirement, driven by synthesis or degradation of multiple new proteins. Therefore, to achieve continuous supply of new proteins they are heavily dependent on nutrient availability, including amino acids, and associated metabolic pathways (71). Although T cell activation in response to antigenic exposure is driven by a multitude of factors ranging from the immune receptor, signaling proteins, cytokines, transcription factors, growing evidence suggests that the dynamic regulation of cellular metabolome controls T cell proliferation, expansion, differentiation, and contraction (71). For instance, mTOR has been shown to integrate multiple cues (cytokines, environmental signals) and governs the outcome of TCR activation or anergy. Recent studies suggest mTOR as a major metabolic switch activated during TCR and CD28 signaling, which triggers the surface expression of glucose, nutrients particularly amino acid transporters (72, 73).

The amino acid uptake in T cells is predominantly controlled by the SLC7 family of transporters (74). The foremost amino acid transporters expressed by T cells include LAT1 (SLC7A5), ASCT2, and GAT1 that play an important role in T cell metabolism and hence the inflammatory responses (75). Also, it has been reported by Sinclair et al. that the leucine transporter, SLC7A5 null T cells are unable to either evoke a robust response to antigen exposure or differentiate into effector cells (76). Moreover, the expression of amino acid transporters is increased upon any antigen exposure and it has been established that the type of amino acid transporter could decide the fate of T cell differentiation into different T cell populations (76). Expression of transporters, LAT1 and ASCT2, and subsequent activation of mTOR pathway tilts the T cell differentiation toward a Th1/Th17-mediated response, which was further confirmed by ASCT2 KO mice that show defective mTORc1 activation and hence, impaired CD4^+^ T cell responses (77–79).

In a separate investigation, GCN2-mediated pathway involvement in T cells proliferation and differentiation has also been reported, where it has been shown that the GCN2 activation has a negative impact on T cells proliferative capacity and affects the Treg cells differentiation (80). Further, a link has also been reported between indoleamine 2,3-dioxygenase (IDO), a tryptophan catabolizing enzyme, regulated T cell responses and immune tolerance with subsequent activation of GCN2 kinase-mediated ISR pathway (80). A recent study reported that topical exposure of phorbol myristate acetate causes plasmacytoid dendritic cell (pDCs) induction in local draining lymph nodes to express IDO, which confers T cell suppressive activity, thereby favors tumor development after carcinogen exposure. Further mechanistic insights depict that IDO expression in pDCs is dependent on inflammatory signaling including interferon-I and II (IFN-I/II) receptors, IL-1/TLR signaling (81). Although depletion of tryptophan locally is known to create an immunosuppressive environment for tumors, GCN2 activation in T cells has no role to play toward tumor immunity (82). Tryptophan depletion and GCN2 activation associated induction of autophagy has also been observed in case of kidney transplantation, where it is known that IFNγ is the central regulator of rejection upon transplantation. The study by Fougeray et al. demonstrates that IFNγ causes tryptophan depletion and thus, controls the immune response toward transplantation, by activating GCN2 and the downstream autophagy (83). Moreover, as the IDO-dependent tryptophan catabolism mimics the condition of amino acid deficiency (80, 84), it has been reported in inflammatory macrophages that the IDO increases IL-10 expression and simultaneously decreases IL-12 levels *via* translation inhibition in a GCN2 dependent manner, which has also been confirmed by the absence of immune-tolerance on GCN2 deletion (46). Macrophages expressing IDO reduce tryptophan in microenvironment, and thus, restrict the CD8^+^ T cell proliferation (65). IDO has also been shown to induce GCN2-dependent immune tolerance to apoptosis antigens. During apoptosis or apoptosis antigen exposure macrophages decrease the proinflammatory cytokine expression posttranscriptionally by inhibiting the IL-12 mRNA association with polysomes while increasing IL-10 transcripts translation (61). Thus, IDO-mediated T cell regulation has been implicated in different inflammatory conditions, but it requires further investigation to elucidate the upstream and downstream pathways involved.

In a separate study, the GCN2 arbitrated T cell response has been associated with antigen presentation and subsequent CD8^+^ immune response to yellow fever vaccine (YF-17D) (85). Yellow fever vaccine is one of the most potent vaccines ever developed in humans. Despite its efficiency and extensive use, the mechanisms implicated in YF-17D-induced protective immune responses remain poorly defined. In an earlier investigation using systems biology approach, it was highlighted that there is an association between the CD8^+^ immune response in blood upon YF17D vaccination and the activation of GCN2 (86). Consequently, in the study by Ravindran et al., the authors investigated the role of GCN2 in adaptive immunity upon YF17D vaccination, where they report that the stimulation of dendritic cells (DCs) with YF17D induces SGs formation, and activates autophagy in a GCN2 dependent manner. The study also confirmed that induction of autophagy enhanced antigen cross-presentation in the YF17D vaccinated mice (85).

**Figure 3:**
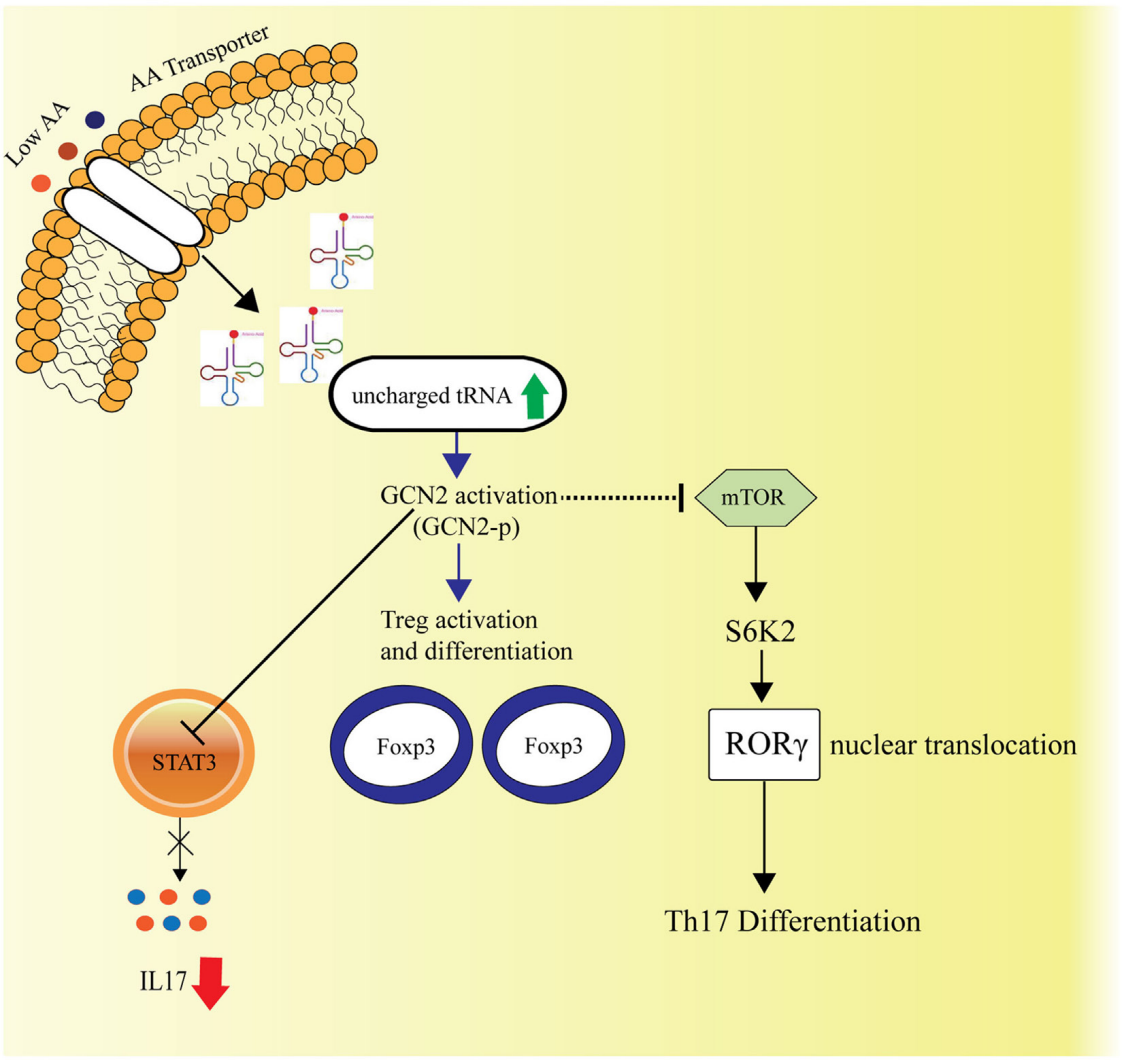
Amino acid sensing and cellular Th17-immune responses. General control nonderepressible-2 kinase (GCN2)-mediated amino acid sensing is also known to dampen the Th17 immune responses by inhibiting the STAT3 transcription factor, essential for Th17 response and simultaneously by hindering the mTORC1 activity, which is known to facilitate Th17 differentiation *via* S6K2 activation followed by nuclear translocation of RORγ. The GCN2 activation also leads to activation and differentiation of the regulatory T cells.

Th17 immune responses among the varied immune effector functions have been concomitant with autoimmune diseases. The naïve CD4^+^ T cells upon stimulation with cytokines IL6, IL1β, TGFβ, result in expression of RORγt and STAT3, the transcription factors, which induce IL17a production and elicits the Th17 response. Carlson et al., in their study investigated the role of AAR pathway on the immune response, where it was observed that AAR pathway activator Halofuginone (HF) dampens the Th17 response, influences STAT3 posttranscriptionally and hence, blocks its expression (87) (Figure 3).

It becomes evident that the amino acid regulation is crucial during T cell immune responses. Therefore, targeting nutrient sensors and amino acid transporters could be an approach to regulate T cell activation and immune response during diseases and thus, to design translational therapeutics.

## Regulation of Amino Acid Signaling During Metabolic and Autoimmune Diseases

Growing evidence categorically suggest a close association between the cellular metabolism and immune response, therefore, it becomes imperative to tightly control nutrient sensing mechanisms to maintain cellular homeostasis. The malfunction of nutrient sensing pathways has been linked with different metabolic diseases in recent times, including obesity, diabetes, atherosclerosis, inflammatory bowel diseases (IBDs), autoimmune diseases, and others, which pose a huge burden on global health (3, 88–90).

The prevalence of obesity and its associated metabolic diseases, such as diabetes, increases enormously worldwide (91–93). In addition to metabolic alterations, both obesity and diabetes are associated with inflammation as well, which led to the notion of the term “meta-inflammation” or metabolically triggered inflammation (94). With nutrient signaling pathways being all interconnected and associated with insulin signaling, it has been reported that the levels of branched-chain amino acids (BCAA) affect the nutrient signaling and metabolism (95, 96). mTOR being a central regulator of amino acid and insulin signaling (97, 98), under amino acid abundance, it phosphorylates IRS1 (at serine 307) and inhibits the downstream insulin signaling (99, 100). Whereas the amino acid deprivation can increase insulin sensitivity, where Xiao et al. reported the activation of GCN2 and simultaneous inhibition of mTOR upon leucine deprivation *in vivo* as well as *in vitro*, which improved the insulin activity under insulin-resistant conditions typically observed in diabetes (11). Furthermore, a recent study unveiled that increased insulin sensitivity is associated with the rise in serum BCCA and decreased BCAA catabolism concomitantly with enhanced proinflammatory gene expression in adipose tissue (101).

Autoimmune diseases, such as rheumatoid arthritis, multiple sclerosis (MS), psoriasis, have been associated with immune responses, particularly with the Th17-mediated responses. Under normal physiology, cells have their own mechanisms to control immune responses and hence, prevent autoimmunity, by induction of apoptosis or through immune tolerance toward self-antigens. The apoptotic cells initiate an anti-inflammatory response to prevent any further development of autoimmunity. In a study on lupus mice, Ravishankar et al. have reported an increase in immune responses and overall mortality post-GCN2 depletion (61). Systemic Lupus Erythematosus is an autoimmune disease where apoptotic cell components act as the antigens to increase cellular immune responses. The authors demonstrated that the IDO in macrophages influences the cytokine levels in a GCN2-dependent manner. It was observed that the anti-inflammatory IL-10 responses are elevated while the IL-12 levels are reduced (61). The role of GCN2 in T cell response has also been identified with the neuronal chronic inflammatory condition, MS (82). MS is characterized by infiltration of Th1 and Th17 cells in CNS, leading to demyelination. It has been reported that during the recovery phase of the disease, the Tregs migrate to the affected area in the CNS and play an important role in keeping MS and inflammation under check. However, the GCN2-depleted Tregs are unable to migrate to the concerned area (82). In another study by Orsini et al., the authors investigated in the GCN2-KO mice subjected to myelin oligodendrocyte glycoprotein peptide and reported higher inflammation and Th1/Th17 cells in the CNS (102). Psoriasis is an autoimmune inflammatory skin disease, associated with CD4^+^ T cell responses (103). In a preliminary study on keratinocyte differentiation, Collier et al. have reported the role of GCN2 phosphorylation-mediated translational control in the normal differentiation and formation of the epidermis. It was observed that the absence of GCN2 disturbed this control over differentiation and therefore could be a cause of various skin diseases, including psoriasis (104).

Amino acid sensing pathways also have control over intestinal inflammation, where the study by Ravindran et al. has demonstrated the role of GCN2 pathway in IBDs (12). Since intestine is the constant environment of fluctuations in nutrient levels, the amino acid sensing becomes crucial to maintain the homeostasis. The authors revealed that induction of DSS-mediated colitis in the absence of GCN2 increased inflammation and subsequent production of inflammatory mediators, such as IL-1β and IL-17. GCN2 activation in macrophages and DCs during IBD results in autophagy activation, which is known to decrease the proinflammatory cytokine production (12, 105, 106). Different studies indicate that the amino acid signaling pathways could be exploited for health benefits. Dietary or calorie restriction, since a long time, is known to have a positive influence on health, stress resistance, and longevity, as it is evident from different studies across species from yeast to rodents (107, 108). The study has shown that proteins demonstrate utmost impact upon dietary restriction (109). It has also been established in humans that the low-protein diet is associated with protection from cancer and diabetes (110). In another study, it has been demonstrated in *Drosophila* that the withdrawal of EAAs contributes more toward the beneficial effect of dietary restriction as compared to the carbohydrates (111, 112). The study by Peng et al. demonstrates dietary tryptophan depletion decreases inflammation and extends protective role in hepatic ischemic injury (14). The evolutionarily conserved GCN2-mediated ISR pathway responds to dietary starvation and is known to modulate immune responses (58). These different studies highlight the health benefits of dietary or amino acid restriction. Concurrently, pharmacological activators like HF can also mimic amino acid starvation, and various studies have reported their immunomodulating anti-inflammatory impact (87). HF is an analog of plant-derived compound febrifugine, which is a known GCN2 activator and has shown to activate amino acid starvation response pathway. It acts by imitating the amino acid proline restriction by competitively binding to and inhibiting the prolyl-tRNA synthetase, leading to accumulation of uncharged prolyl-tRNA and further initiation of AAR pathway (47). In a separate study by Sundrud et al., the authors have also demonstrated in a mouse model of experimental autoimmune encephalomyelitis (EAE) or MS that HF targets precisely the proinflammatory Th17 response by disrupting the STAT3 phosphorylation (113). HF has been tested in different disease conditions, including malaria, cancer, and autoimmune diseases, with a few clinical trials in the field (114). Consequently, more studies with an emphasis on the mechanism of action of HF in amino acid sensing and the downstream responses could help us in designing therapeutics that could be tested in the different conditions discussed earlier.

## Conclusion

Although the association between nutrient sensing with immune response and the concept of “immunometabolism” is not new, yet there has been a lack of mechanistic understanding, which requires further investigations. Several studies illustrate the importance of amino acid sensing in evoking the innate as well as adaptive effector immune functions. For instance, recent studies have established the link between amino acid starvation sensor GCN2 and its control over inflammation by highlighting the posttranscriptional mechanisms, and autophagy. These studies not only raise some important questions with respect to metabolic control of Th17/Treg ratios and immune response but also point out that it might be interesting to test these hypotheses in the case of obesity as well as during inflammation observed in various diseases including neurological disorders. Therefore, all these reports and preliminary investigations open up avenues for future research in cross-talk between the nutrient sensing and immune responses, which can be extrapolated to clinical trials for inflammatory and metabolic diseases.

## Author Contributions

NK conceptualized the idea. SB, GM, and NK wrote the manuscript. AM made the figures.

## Conflict of Interest Statement

The authors declare that the research was conducted in the absence of any commercial or financial relationships that could be construed as a potential conflict of interest.
